# A Review of National Level Guidelines for Risk Management of Cardiovascular and Diabetic Disease

**DOI:** 10.7759/cureus.26458

**Published:** 2022-06-30

**Authors:** Ramesh Pandit, Trupti Pandit, Lokesh Goyal, Kunal Ajmera

**Affiliations:** 1 Medicine, Penn Medicine Chester County Hospital/University of Pennsylvania, Philadelphia, USA; 2 Hospital Medicine/Pediatrics, Inspira Medical Center, Vineland, USA; 3 Hospital Medicine, CHRISTUS Spohn Hospital Corpus Christi - Shoreline, Corpus Christi, USA; 4 Epidemiology, George Washington University, Washington, USA

**Keywords:** hypertension, ascvd, counseling for cardiovascular disease risk reduction, gestational diabetes, risk factor management, national guidelines, prevention, prediabetes, diabetes, cardiovascular disease

## Abstract

Cardiovascular diseases and diabetes are among the leading preventable causes of morbidity and mortality. Cardiovascular disease risk reduction aimed to address the significant modifiable risk factors, including lifestyle-related risk factors, hypertension, hyperlipidemia, and diabetes. Given the severity and disease burden, many insurances, including Medicare, cover the annual counseling for risk reduction of cardiovascular disease. Although numerous national-level guidelines are available for managing these conditions, most of them focus on disease management. Given the broad areas covered in these recommendations, a concise review summarizing the measures addressing the preventive approach in these conditions is not readily available. Herewith, we review and outline the currently available guidelines from national-level publications with principal attention to the primary prevention measures to provide a broad overview and assist providers with the risk reduction counseling.

## Introduction and background

Background

Atherosclerotic cardiovascular disease (ASCVD) is a group of disorders that comprises acute coronary syndromes (ACS), myocardial infarction (MI), angina, coronary or other arterial diseases, stroke or peripheral arterial disease, transient ischemic attack (TIA), which are considered to be of atherosclerotic origin. According to the United States Preventive Services Task Force (USPSTF), cardiovascular disease (CVD) has been reported to cause one of every three adult deaths [1]. CVD and diabetes are among the top modifiable and non-modifiable risk factors for increased overall morbidity and mortality [2]. Modifiable risk factors include hyperlipidemia, smoking, hypertension, obesity, and diabetes. Non-modifiable include a family history of CVD before 50 years of age in male relatives or 60 years of age in female relatives. Management of these risk factors falls under the primordial and primary prevention, with primordial prevention focusing on influencing population-based healthy lifestyle choices. In contrast, primary prevention aims to delay or prevent the onset of the disease. The USPSTF recommends annual counseling for risk reduction of cardiovascular diseases for all adults with risk factors [3].

Epidemiology

The prevalence of hypertension alone in 2010 was estimated to be 31.1% of adults worldwide [4]. The prevalence of hypertension from 2011 to 2014 was 45.6% in adults as per 2017 American College of Cardiology (ACC)/American Heart Association (AHA) and 29.9% as per the 2014 Joint National Committee (JNC) 8 in the US, posing a substantial financial burden on patients and the healthcare system [5]. These further highlight the importance of risk reduction for ASCVD and diabetes. Despite the numerous initiatives, the global risk and prevalence of diabetes and hypertension continue to increase, with a higher increase in lower-income countries [6,7].

The overall prevalence of prediabetes in adults aged ≥20 years was 34.4%, while diabetes prevalence was estimated to be 12.5%, based on the NHANES 2011-2014 data [8]. According to the American Diabetes Association (ADA) [6], diabetes affects one out of every 10 persons aged 20 to 79 (about 537 million people globally). As of 2021, 361 million persons aged 20 to 79 in the United States have been diagnosed with diabetes. This number is predicted to be one in every nine people by 2030, and one in every eight people by 2045, with over 400 million by 2045 in the United States, while worldwide, that number is expected to be close to 1 billion by 2045 [9]. 

Data collection methodology

A literature search was performed with MEDLINE for Publications between the years 2010 and 2021. The investigation was restricted to national-level guidelines from the United States, Canada, Europe, and international guidelines written in English. The search syntax included the following terms, “Cardiovascular disease,” “Diabetes,” “Prediabetes,” “Prevention,” “Guidelines,” “Hyperlipidemia,” “Hypertension,” “Blood pressure,” “Risk,” and “Diagnosis.” We reviewed the referenced articles and professional specialty association’s publications for further clarification, and commentary regarding these guidelines. The publications were obtained, by two physician researchers (RP and KA) separately, and reviewed by all authors. The recommendations addressing cardiovascular and diabetes prevention, and risk reduction, are summarized and structured below for simplicity of comprehension.

## Review

Risk assessment and screening recommendations

The USPSTF and the European Society of Cardiology/European Atherosclerosis Society (ESC/EAS) recommend screening all adults for cardiovascular risks [10,11]. Fundamental principles behind the risk assessment and screening are that most CVD is preventable by addressing well-defined risk factors with effective risk management therapies. Even for patients with no known diagnosis of hypertension, diabetes, hyperlipidemia, or cardiovascular disease, Diet, physical activity, and tobacco use should be discussed and addressed in each annual preventive care. A team-based approach towards prevention should be followed, as appropriate and prompt management of the risk factors is paramount in preventing complications and optimizing health outcomes.

Calculating the Risks

The ACC/AHA ASCVD risk calculator is a valuable tool for gauging the 10-year risk of a first ASCVD event (available online at tools.acc.org/ASCVD-Risk-Estimator-Plus) [12]. The calculator incorporates age, diabetes, sex, race, tobacco usage, dyslipidemia, systolic blood pressure, and presence of hypertension, as detailed in Table 1. Providers should assess the 10-year risk of a first ASCVD event to classify ASCVD risk better and help guide therapy. The risk estimator also reports lifetime risk, which is useful for those <50 years of age. The 30-year cardiovascular disease risk calculator based on the Framingham Heart Study, which adds body mass index (BMI) as an extra risk factor to those listed above, is the other most often used tool [13]. The current risk calculators ignore diabetic complications and the duration of diabetes. The overall risk prediction in persons with and without diabetes is not significantly different [10,14], validating the use of risk calculators in people with diabetes.

**Table 1:** Key features of ASCVD risk calculator by American College of Cardiology/American Heart Association Atherosclerotic cardiovascular disease (ASCVD), Coronary Artery Calcium Score (CAC), Chronic Kidney Disease (CKD)

Access to the tool	Available online at tools.acc.org/ASCVD-Risk-Estimator-Plus
Risk Assessment	Estimates 10-year risk (age 40-75 yrs); and lifetime risk of ASCVD for younger patients (age 25-59 yrs)
Key Points Considered	Age, Sex, Diabetes, Race, Tobacco usage, Dyslipidemia, Hypertension (systolic blood pressure)
Risk Factors/ enhancers	Family history of premature ASCVD; Cholesterol levels (LDL-C ≥ 160 mg/dL; Triglycerides ≥175-499 mg/mL); CKD; metabolic syndrome; Ethnicity; Inflammatory markers.
Categories	low (<5%), borderline (5% to <7.5%), intermediate (≥7.5% to <20%), or high (≥20%) 10-year risk
Fixed Outcome	Clinician and Patient discussion regarding risk factors and lifestyle modifications.
Role of ASCVD score in patient care	Guide the decision regarding statin therapy and the intensity of the therapeutic measures
Role of Coronary Artery Calcium Score (CAC)	Those with intermediate or select borderline risk can use the CAC score to guide the decision process further.

The 2013 American College of Cardiology (ACC) and American Heart Association (AHA) hypertension guidelines recommend those aged 40 to 75 can be categorized as <10% or >10% 10-year ASCVD risk for therapeutic decisions [15]. As per the 2018 Cholesterol Guidelines [12], adults should be classified as having low (<5%), borderline (5% to <7.5%), intermediate (≥7.5% to <20%), or high (≥20%) 10-year risk, which can further help determine the treatment intensity.

Per the 2019 ESC Guidelines [11], patients with known CVD, end-organ damage, three major risk factors, or an early-onset type 1 diabetes mellitus were at a very high risk of developing ASCVD. The high-risk group was defined as those with diabetes mellitus for >10 years and at least one additional risk factor (but no evidence of end-organ damage). Furthermore, patients with type 1 diabetes mellitus younger than age 35 or those younger than 50 years of age for type 2 diabetes mellitus have diabetes mellitus duration <10 years and lack other CV risk factors was defined as a moderate risk group for developing ASCVD [11].

Recommendations for the management of the individual risk factors

Identifying the modifiable risk factors for ASCVD is the key to prevention and risk reduction. The 2019 guidelines by ACC/AHA on the Primary Prevention of Cardiovascular Disease [2] also focus on addressing the essential risk factors, and encouraging a healthy lifestyle throughout life is the crucial goal (Table 2).

**Table 2:** Summary of risk reduction recommendations for addressing cardiovascular and diabetes risk factors Atherosclerotic cardiovascular disease (ASCVD)

Risk Factor	Recommendation
Diet	Limit the food higher in added sugars, saturated fat, and sodium alcohol consumption
Physical Activity	Sit less and move more. Follow the recommended duration/week of physical activity based on age and ability.
Overweight and Obesity	Behavioral Counseling, Calorie restriction, and Physical activity
Tobacco use	Strongly encourage, and advise to quit.
Hyperlipidemia	Statin and Non-statin therapies based on Cholesterol levels and ASCVD risk score
Hypertension	Age-based screening and treatment goals individualized to each patient
Prediabetes, and Diabetes	Lifestyle modification, Diet and weight control, and Pharmacological intervention based on risk factors

Diet

The Dietary Guidelines for Americans 2022-2025 version by the US Department of Agriculture (USDA) has made the following dietary recommendations to prevent ASCVD [16]. Patients are recommended to follow a healthy eating pattern across the lifespan to satisfy adequate nutrient requirements, help acquire healthy body weight, and reduce the risk of chronic disease. A healthy dietary practice is to take in nutrient-dense food and beverage at recommended amounts with choices personalized to individual preferences and needs while maintaining calorie limitations, as this will benefit all persons regardless of age, race, ethnicity, or present health state. It is advised to limit the intake of foods that are higher in added sugars, saturated fat, and sodium, including a limit on the intake of alcoholic beverages. The goal is for added sugars to amount to less than 10% of calories consumed per day; saturated fat to amount to less than 10% of calories consumed per day; sodium intake to be limited to less than 2,300 milligrams per day or even less for those younger than age 14 years. There is a shift in guidelines to consider cultural and personal preferences to make healthier food and beverage choices easier to accomplish and maintain. The recommended daily allowance for alcohol consumption is two standard drinks for men and one and a half for women (10 g alcohol/standard drink).

Physical activity

The 2018 Physical Activity Guidelines for Americans by the Department of Health and Human Services (HHS) are based on the 2018 Physical Activity Guidelines Advisory Committee Scientific Report [17]. The WHO 2020 guidelines on physical activity [18] also include similar recommendations. The benefits are proportional to the inactivity level before the change and are directly proportional to the amount of physical activity change. The most significant emphasis is on moving more and sitting less [19]. The age-based recommendations include a) Children and adolescents aged six through 17 years: 60 minutes or more of moderate-to-vigorous physical activity daily. b) Adults: 150 to 300 minutes of moderate-intensity physical activity per week, or 75 to 150 minutes of vigorous-intensity aerobic physical activity per week, or an equivalent combination, with muscle-strengthening activities on two or more days as part of the weekly goals. c) Older adults: Focus on different components of physical activity, including balance training and aerobic and muscle-strengthening activities. Those pregnant or other factors limiting activity have revised recommendations. Pregnant and postpartum women should do at least 150 minutes of moderate-intensity aerobic activity weekly. Those with chronic conditions or disabilities: if able, should follow the guidelines as above, or as much of the same, with limitations.

Obesity and weight loss

For overweight and obese patients, caloric restrictions and counseling is recommended for achieving and maintaining weight loss. The 2019 ACC/AHA guidelines, for all patients with BMI < 35 kg/m^2^, are recommended to monitor waist circumference, as an increased waist circumference has been associated with increased ASCVD risk [2].

Tobacco use

As per the 2019 National Health Interview Survey, an estimated 18.4% of adults in the United States smoked, making it the leading cause of preventable mortality [20]. Electronic cigarettes are adolescents' most prevalent tobacco product [21]. All adult patients should be assessed for tobacco use at every healthcare visit, as tobacco Smoking and other uses (e.g., chewing tobacco) and even secondhand smoke increase the risk of all-cause mortality and cause ASCVD [10]. Even secondhand smoke is a cause of ASCVD. The USPSTF recommends that All Patients who use tobacco should be strongly encouraged and advised to quit [1].

Prediabetes and diabetes

Prediabetes is defined as random plasma glucose levels are between 140 mg/dL and 199 mg/dL and an impaired fasting plasma glucose level of 100-125 mg/dL, two-hour glucose tolerance test with blood glucose levels between 140 mg/dL to 199 mg/dL or hemoglobin A1C levels between 5.7% and 6.4% [22]. Prediabetes is a risk factor for the development of type 2 diabetes and is also associated with increased micro and macrovascular complications [23]. Per the ADA 2019 guidelines, annual monitoring for the development of type 2 diabetes is recommended for those with prediabetes. Prediabetes is associated with an increased risk of cardiovascular disease and all-cause mortality [24]. Approximately 32.2% of people with diabetes develop CVD as a complication [5].

Prevention and treatment are focused on lifestyle modification and weight control in pre-diabetics. The US Diabetes Prevention Program Outcomes Study has shown a 34% reduction in diabetes conversion at ten years. The goal of lifestyle modifications is to achieve and maintain a 7% loss of initial body weight and to target moderate-intensity physical activity for at least 150 min/week. Metformin therapy should be considered in those with prediabetes with additional risk factors, e.g., BMI ≥ 35 kg/m^2^, age < 60 years, and history of gestational diabetes mellitus. Bariatric surgery is another effective way of preventing T2 diabetes mellitus in patients with prediabetes and BMI above 35 kg/m^2^. As per the ADA, diabetes is classified into four categories, as in Table 3 [25].

**Table 3:** Diabetes classifications

Diabetes Categories	Pathophysiology
Type 1 diabetes	Immune-mediated or idiopathic
Type 2 diabetes	Variable combination of insulin resistance and insulin secretory defect/deficiency.
Specific types of diabetes due to other causes	Genetic defects in β-cell function, or those affecting insulin receptors, diseases of the exocrine pancreas, e.g., cystic fibrosis and pancreatitis), drug- or chemical-induced diabetes, and others.
Gestational diabetes mellitus	impaired glucose tolerance due to pancreatic β-cell dysfunction, in addition to chronic insulin resistance

The diagnostic criteria for diabetes include a) fasting blood glucose ≥126 mg/d on one or two separate occasions, b) the presence of classic symptoms of hyperglycemia, and random plasma glucose ≥200 mg/dL, c) 2-h Postprandial Glucose >200 mg/dL (11.1 mmol/L) during oral glucose tolerance test or d) hemoglobin A1c ≥6.5% [25,26].

Recommendations for gestational diabetes mellitus include screening for undiagnosed diabetes or prediabetes on the first prenatal visit using standard criteria. ADA recommends screening for gestational diabetes at 24-28 weeks of pregnancy in women who have not been previously diagnosed with diabetes [27]. Those with a history of gestational diabetes mellitus should undergo screening for prediabetes or diabetes using the 75-g oral glucose tolerance test at 4-12 weeks postpartum, followed by lifelong screening for diabetes at least every three years [27].

Numerous risk assessment tools for the development of diabetes are available online and in paper forms. However, they lack appropriate validation and are not part of any guidelines. ADA's risk test includes age, sex, race, family history, hypertension, dyslipidemia, physical inactivity, obesity, and gestational diabetes [28]. The screening questions can be utilized to look for different risk factors that can increase one's risk for diabetes.

Diet counseling for prediabetes and diabetes should include discussion regarding glycemic index and glycemic load. The glycemic index is a scale that classifies carbohydrate-containing foods based on their effect on blood sugar levels. The three glycemic index ratings are low (55 or fewer), medium (56 to 69), and high (70 or more). The limitation of the glycemic index is that it does not consider the amount of food consumed. The glycemic load rating measures how carbohydrate affects blood sugar level, considering the quantity of food and the glycemic index. A low glycemic index diet contains most fruits, green vegetables, beans, lentils, steel-cut oats, whole-grain food, seafood, nuts, olive oil, herbs, and spices. A medium glycemic index diet incorporates banana, sweet corn, raisins, instant oats & breakfast cereals.

In contrast, the high glycemic index diet contains white bread, white rice, potatoes, cakes, and cookies. A high glycemic index is linked to a higher risk of death from cardiovascular events in people with and without heart disease [29]. A low glycemic diet can also help with weight loss and reduce the risk of heart disease [30].

Among the pharmaceutical measures, Metformin reduced the risk of diabetes progression by 31% in patients with prediabetes compared to placebo. It was found to be particularly beneficial for patients with obesity, higher fasting glucose levels, or a history of gestational diabetes [31]. Metformin improves lipoprotein subtraction, C reactive protein, and tissue plasminogen activator and reduces the incidence of metabolic syndrome by 17% [32].

Buse et al. [33] summarized the recommendations from the 2019 update from the ADA and the European Association for the Study of Diabetes, especially concerning Glucagon-like peptide 1 (GLP-1) receptor and sodium-glucose cotransporter 2 (SGLT2) medications. For patients with and without type 2 diabetes, GLP-1 receptor agonist and/or SGLT2i therapy is recommended for individuals with a high risk of developing ASCVD; and to reduce progression to chronic kidney disease (CKD). The 2019 ESC Guideline also suggested using SGLT-2 inhibitors and GLP-1 agonists as first-line treatment for high or very high CV risk patients instead of Metformin and added to those already on Metformin.

The SGLT2i class has demonstrated usefulness in reducing CVD, heart failure (HF) related hospitalization, and cardiovascular death in people with and without the pre-existing cardiovascular disease [34]. It has also been associated with decreasing albuminuria [35]. The Canadian Cardiovascular Society HF guidelines recommend using SGLT2i to prevent and treat HF patients with and without type 2 diabetes [36].

Hyperlipidemia

Lipid profile tests should be performed initially at the time of diagnosis, and individuals under 40 should have them repeated at least every five years. Depending on the cholesterol levels, older patients may require more frequent lipid profile testing. Reduced saturated and trans-fat intake and increased plant stanols/sterols, n-3 fatty acids, and viscous fiber (found in citrus, oats, and legumes) are all recommended for lowering dyslipidemia. Controlling blood sugar levels can also help patients with excessive triglycerides and poor glycemic control improve their plasma lipid levels [37].

Hypertriglyceridemia should be treated with dietary and lifestyle modifications such as weight loss, alcohol abstinence, and treatment of secondary causes such as avoidance of triglyceride-raising medications. Severe hypertriglyceridemia may require pharmacologic therapy such as fabric-acid derivatives and fish oil. According to the ACC/AHA guidelines on Cholesterol Management [2,12], the 20-39 years age group should be encouraged to promote a healthy lifestyle unless having high LDL-C (≥160 mg/dL), for which addition of drug therapy to the lifestyle modifications is recommended. In those from 40 to 75 years of age, the 10-year ASCVD risk assessment should be followed by a discussion regarding therapeutic measures. Those at higher estimated risk are likely to receive more benefits from initiating statin therapy. For those above 75 years of age, physician and patient discussion regarding risk and benefit evaluation should happen before making a final decision regarding statin treatment. Once a patient is started on statins, cholesterol levels can be checked in one to three months and after dosage adjustments. High-intensity statin therapy will reduce LDL by approximately 50% or more, and moderate-intensity statin medications will achieve a 30 to 49% reduction in LDL cholesterol.

ACC/AHA guidelines for the years 2018 and 2019 for ASCVD prevention include the following key recommendations: a) Age-group 40 to 75 years with diabetes mellitus are considered intermediate to high risk and are recommended for statin therapy; b) ASCVD risk score calculation guides with statin therapy as well as the intensity of the same; c) adult diabetics without a history of CVD are recommended to use a low- to a moderate-dose statin to prevent CVD events and mortality benefits; d) For those with uncertain risk assessment, a coronary artery calcification score can be helpful in refining risk assessment. European guidelines for the primary prevention of ASCVD concur with ACC/AHA in this regard [38]. e) Those with severe primary hypercholesterolemia (i.e., LDL-C level ≥190 mg/dL or 4.9 mmol/L) begin high-intensity statin therapy, irrespective of the 10-year ASCVD risk. The maximum tolerated statin dose should be used for patients who do not take the expected intensity of the statin medications [39]. The addition of non-statin therapies, such as ezetimibe, or newer medicines for patients at very high risk of ASCVD, especially with persistent hypertriglyceridemia, has been recommended by 2021 ACC guidelines [40].

Hypertension

Hypertension is a substantial risk factor for the development of ASCVD. Every routine appointment should include a blood pressure check for all adults. According to the 2021 USPSTF guideline, for adults aged 18-39 years, without risk factors or prior history of HTN, routine screening every three to five years is warranted. Adults with an increased risk of hypertension or age>40 should have an annual screening [41].

Blood pressure should be measured by a certified professional. It should adhere to the following principles for the public: After 5 minutes of rest, measure in a seated posture with feet on the floor and arm supported at heart level. The diagnosis is based on an average of ≥2 readings obtained on ≥2 separate occasions. Masked hypertension, white-coat hypertension, or other disparities between the office and “real” blood pressure can be detected using home blood pressure self-monitoring and 24-hour ambulatory blood pressure monitoring. According to the 2017 guidelines by the American College of Cardiology (ACC) and the AHA, hypertension is classified in stage 1 (systolic pressure 130-139/diastolic 80-89 mm Hg) and stage 2 (systolic pressure ≥ 140 or diastolic ≥ 90 mm Hg), while the term pre-hypertension is replaced by elevated blood pressure (systolic pressure 120-129, diastolic < 80 mm Hg) [42].

Epidemiological study analysis has shown that blood pressure > 115/75 mm Hg is associated with an increase in cardiovascular event rate and mortality in individuals with diabetes [43]. JNC 8 guidelines, compared to prior JNC 7 guidelines, changed the systolic blood pressure goals from the previous <140/90 mm Hg goal for most patients and <130/80 mm Hg goal for patients with hypertension and significant comorbidities [44]. The patients aged 60 years or older without diabetes or chronic kidney disease, the goal blood pressure level is <150/90 mm Hg. The patients aged 18 to 59 years, with diabetes or chronic kidney disease (CKD), or without significant comorbidities, and patients 60 years or older, the goal blood pressure level is <140/90 mm Hg.

Individualized blood pressure objectives should be considered based on activity level, frailty, dependency, and tolerance. The 2020 International Society of Hypertension Global Hypertension Practice Guidelines are identical to the recent US and European guidelines recommending a goal BP of <130/80 (preferably <120/70 mm Hg if tolerated) in patients younger than 65. For patients older than 65 years, aim for BP < 140/90 mm Hg if tolerated.

Lifestyle Modification for Blood Pressure Control

Initial treatment should focus on non-pharmacological intervention such as lifestyle modifications as initial therapy for all adults newly diagnosed with elevated blood pressure or stage 1 hypertension (130 to 139/80 to 89 mm Hg). In contrast, lifestyle modifications and medications are recommended for those with existing CVD or increased CVD risk. The Dietary Approaches to Stop Hypertension (DASH) study assessed the effect of a healthy diet in individuals without diabetes and has shown its antihypertensive effects like those of pharmacologic monotherapy. Key points to address include reducing excess body weight through dietary caloric restriction, restriction of dietary sodium intake to <2,300 mg/day, increasing consumption of fruits and vegetables (8-10 servings per day) with low-fat dairy products (2-3 servings per day), avoiding excessive alcohol consumption (no more than two servings per day in men and no more than one serving per day in women), and increasing physical activity levels [44,45].

Pharmacologic Interventions for Blood Pressure Control

Reducing blood pressure with regimens based on various antihypertensive medications, including ACE inhibitors, angiotensin receptor blockers (ARBs), diuretics, and calcium channel blockers, can reduce cardiovascular events [46]. Medication selection is based on the patient's race, the occurrence of adverse effects, and personal preferences. Detailed review and discussion regarding the pharmacological interventions for treatment purposes were out of scope for this article and, therefore, have not been included here.

Role of anti-platelet medications for the prevention of ASCVD

Per the USPSTF recommendations, Aspirin is recommended for men aged 45 to 79 years and for women aged 55 to 79 years to reduce the risk of MI recurrence when the potential benefit from a reduction in MI outweighs the potential harm due to an increase in the risk of gastrointestinal bleeding. In contrast, 2019 AHA guidelines do not recommend the routine use of Aspirin for primary prevention due to a lack of benefits when balanced against risks. For patients with ASCVD and documented aspirin allergy, clopidogrel (75 mg/day) should be used. Dual antiplatelet therapy (with low-dose Aspirin and a P2Y12 inhibitor) is reasonable for a year post-ACS. It may have benefits beyond this period, and these can be considered secondary prevention therapies for patients with known complications related to ASCVD.

## Conclusions

CVD has substantial health and economic ramifications in the United States and around the globe. Given the numerous common risk factors, anyone diagnosed with hypertension or diabetes should be assessed for all other cardiovascular risk factors, and intervention should begin as soon as possible. Those without any known diseases or risk factors should undergo risk assessment annually, in form of questionnaires, or virtual or in-person visits. Many cardiovascular and diabetic risks are intertwined. Primordial prevention strategies combine biological and environmental factors with critical elements, including a healthy diet, a healthy weight, and addressing other risk factors tailored to the individual.

Guidelines should be utilized for what they are: a guide to aid decision-making, with a team-based approach to cardiovascular risk reduction and early diagnosis of diabetes and hypertension, along with implementing primordial, primary, and secondary preventive interventions. Screening should be done frequently for younger (<50 years of age) patients with one or more clinical manifestations that place them at intermediate to high risk for CVD and diabetes. We opine that the screening tools should be made readily available to the public and promoted, including the use of social media, especially in light of the recent COVID-19 pandemic and lack of timely access to preventive services. Clinicians must also assess patients' economic status, socio-cultural resources, access to services, and health literacy to accomplish protracted improvements in lifestyle habits.
